# Multidisciplinary advances in pancreatic cancer surgery: a scientific report from the 2024 Salerno conference

**DOI:** 10.3332/ecancer.2025.2035

**Published:** 2025-11-14

**Authors:** Dario Cattel, Rosario De Feo, Francesco Sabbatino, Stefano Pepe, Alessandro Puzziello

**Affiliations:** 1 General Surgery Unit, AOU San Giovanni di Dio e Ruggi d’Aragona, University of Salerno, Largo Città di Ippocrate, 84131 Salerno, Italy; 2 Oncology Unit, Department of Medicine, Surgery and Dentistry, University of Salerno, 84081 Baronissi, Italy; a https://orcid.org/0009-0006-4988-0733; b https://orcid.org/0009-0009-9573-5096; c https://orcid.org/0000-0001-6431-8278; d https://orcid.org/0000-0001-6280-8142; e https://orcid.org/0000-0002-1970-7386

**Keywords:** cancer, pancreas, oncology, surgery, pancreatic cancer surgery, multidisciplinary oncology, chemotherapy, minimally invasive techniques, conference

## Abstract

The 'Pancreatic Cancer’ conference, held on 8 November 2024, at the San Giovanni di Dio e Ruggi d’Aragona University Hospital in Salerno, Italy, brought together leading experts in pancreatic surgery and oncology. The event aimed to provide a comprehensive overview of recent developments in the multidisciplinary treatment of pancreatic adenocarcinoma, with particular focus on advanced surgical strategies. Topics included neoadjuvant chemotherapy, surgical planning and techniques (open, laparoscopic, robotic), complex resections and management of postoperative complications. The conference was accredited by the National Continuing Medical Education Program and integrated into the regional 'Campania Pancreas’ initiative. Featuring national and international speakers, including a keynote lecture by Prof. C.R. Ferrone from Cedars-Sinai Medical Centre, the meeting served as a platform for knowledge exchange and innovation in pancreatic cancer care.

## Overview, objectives, context and commemoration

The conference ‘Pancreatic Cancer’ was held on 8 November 2024, at the ‘Aula Scozia’ of the San Giovanni di Dio e Ruggi d'Aragona University Hospital in Salerno. The scientific directors were Prof. Alessandro Puzziello, Director of the UOC Clinical Surgery and Kidney Transplants at the same hospital and Prof. Stefano Pepe, Director of the UOC Oncology Department at the same institution.

The primary objective of the conference was to provide a comprehensive overview of various aspects of pancreatic adenocarcinoma surgery and to examine recent advancements in its multidisciplinary management. The focus was on the most advanced and innovative surgical approaches for pancreatic neoplasms, as well as on discussing areas of uncertainty regarding indications and complications related to pancreatic surgery. The educational objectives were primarily aimed at clinical documentation, diagnostic and rehabilitative care pathways, assistance profiles and treatment protocols.

This conference was part of the National Continuing Medical Education Program of the Ministry of Health for the following professional categories: General Surgeons, Gastroenterologists, Radiologists, Oncologists, Diabetologists and Nurses. Additionally, the meeting was integrated into the ‘Campania Pancreas’ project, a regional platform dedicated to combating pancreatic cancer by providing information, engaging with the public to enhance patient care, promoting oncological research in this field and mitigating medical migration. It was also included in the third mission of the Department of Medicine, Surgery and Dentistry ‘Scuola Medica Salernitana’ at the University of Salerno.

A particularly significant and emotional moment of the event was the commemoration of Cristiano Huscher, an internationally renowned surgeon who tragically passed away. As Prof. Puzziello remarked, Huscher would have animated the conference with his critical insights and vibrant personality, but his legacy endures in his writings, in the hearts of many, and in the skilled hands of numerous surgeons.

## Surgical indications and strategy

The conference commenced with greetings from local authorities, followed by the introduction of the first session by the session chairs and moderators: L. Docimo, F. Sabbatino and G. Conzo.

Dr. P. Erra opened the discussions with a presentation on imaging for assessing pancreatic tumour resectability before and after neoadjuvant chemotherapy (NAC) [1]. Dr. R. Bianco then delved into the topic of NAC for Borderline Resectable Pancreatic Cancer (BRPC) and Locally Advanced Pancreatic Cancer (LAPC), exploring whether NAC could increase postoperative morbidity and mortality [1]. Subsequently, Dr. A. Loffredo provided insights into surgical indications and strategies for BRPC and LAPC, focusing on the concepts of abutment and encasement.

## Surgical planning and techniques

The second session was introduced by session chairs and moderators V. Bottino, C. Molino and F. Izzo. Dr. B. Ielpo initiated the session with a discussion on surgical planning for pancreaticoduodenectomy (PD), addressing when to perform the procedure via open surgery, laparoscopy or robot-assisted techniques. Dr. Malleo then explained the ‘Artery-First Approach,’ emphasising the identification and control of the Superior Mesenteric Artery and other relevant arteries before tumour resection [2]. Dr. P. Addeo shifted the focus to complex resections, particularly PD and vascular resections. Additionally, Dr. A. Coratti shared his experience with robotic pancreatic anastomosis [4], Dr. M. Viola discussed laparoscopic pancreatic anastomosis and Dr. F. Crafa addressed pancreatic anastomosis in open surgery.

A highlight of the conference was the guest lecture by Prof. C.R. Ferrone, who traveled from Los Angeles, where she is affiliated with the Department of Surgery at Cedars-Sinai Medical Centre. She delivered a keynote address on surgical outcomes and patient selection for surgery following NAC [5].

## Postoperative management and conclusion

The final session was chaired and moderated by D. Cuccurullo, A. Maurano, U. Bracale and M. Vitale. Dr. P. Zeppa opened the session with a presentation on the pathological evaluation of surgical margins and the concept of oncological radicality. Dr. A. Belli then addressed strategies for preventing and managing a common postoperative complication, pancreatic fistula [3]. Expanding on this topic, Dr. F. Laurino discussed when the intervention of an interventional radiologist is necessary, while Dr. C. Zulli highlighted the role of endoscopy in managing pancreatic fistula-related complications. Dr. G. Clemente, a diabetologist, provided insights into indications and post-total pancreatectomy management. Finally, Dr. R. Montalti clarified concerns regarding the long-term consequences of pancreatic surgery.

The event witnessed a remarkable level of participation, with an attendance exceeding 200 individuals, reflecting strong engagement from the scientific and medical community. This high turnout highlights the growing interest in pancreatic oncology and the need for continuous discussion on advancements in diagnosis, treatment and patient management.

However, this was not the first time that the Ruggi d'Aragona Hospital hosted a conference dedicated to pancreatic neoplasms. A previous symposium, held on 22 January 2020, under the scientific direction of Prof. A. Puzziello, had also achieved significant success, drawing experts from various disciplines to share their latest research findings and clinical experiences. The recurrence of such events underscores the hospital’s commitment to fostering a collaborative scientific environment, promoting the exchange of knowledge and encouraging multidisciplinary approaches to tackling one of the most challenging malignancies in modern oncology.

In conclusion, the conference underscored the significance of innovative surgical techniques, precise patient selection, effective multidisciplinary collaboration and proactive management of postoperative complications as essential components in improving outcomes and quality of life for patients with pancreatic cancer.

## Conflicts of interest

The authors declare that they have no conflicts of interest related to the content of this manuscript.

## Funding

The authors declare that no funds, grants or other support were received for the preparation of this work.
